# Epstein–Barr Virus Epidemiology, Serology, and Genetic Variability of LMP-1 Oncogene Among Healthy Population: An Update

**DOI:** 10.3389/fonc.2018.00211

**Published:** 2018-06-13

**Authors:** Maria K. Smatti, Duaa W. Al-Sadeq, Nadima H. Ali, Gianfranco Pintus, Haissam Abou-Saleh, Gheyath K. Nasrallah

**Affiliations:** ^1^Biomedical Research Center, Qatar University, Doha, Qatar; ^2^Department of Biomedical Science, College of Health Sciences, Qatar University, Doha, Qatar; ^3^Department of Biological and Environmental Sciences, College of Arts and Sciences, Qatar University, Doha, Qatar

**Keywords:** blood donors, Epstein–Barr virus, LMP-1 oncogene, seroprevalence, transfusion, viremia

## Abstract

The Epstein–Barr virus (EBV) is a DNA lymphotropic herpesvirus and the causative agent of infectious mononucleosis. EBV is highly prevalent since it affects more than 90% of individuals worldwide and has been linked to several malignancies including PTLDs, which are one of the most common malignancies following transplantation. Among all the EBV genes, most of the recent investigations focused on studying the LMP-1 oncogene because of its high degree of polymorphism and association with tumorigenic activity. There are two main EBV genotypes, Type 1 and 2, distinguished by the differences in the EBNA-2 gene. Further sub genotyping can be characterized by analyzing the LMP-1 gene variation. The virus primarily transmits through oral secretions and persists as a latent infection in human B-cells. However, it can be transmitted through organ transplantations and blood transfusions. In addition, symptoms of EBV infection are not distinguishable from other viral infections, and therefore, it remains questionable whether there is a need to screen for EBV prior to blood transfusion. Although the process of leukoreduction decreases the viral copies present in the leukocytes, it does not eliminate the risk of EBV transmission through blood products. Here, we provide a review of the EBV epidemiology and the genetic variability of the oncogene LMP-1. Then, we underscore the findings of recent EBV seroprevalence and viremia studies among blood donors as a highly prevalent transfusion transmissible oncovirus.

## Introduction

The Epstein–Barr Virus (EBV), also called human herpesvirus 4, is a lymphotropic herpesvirus and the causative agent of infectious mononucleosis (IM) (1). It was first discovered in cells isolated from African Burkitt’s lymphoma, later, it was recognized that it is highly prevalent worldwide (2). Similar to other herpesviruses, following a primary infection, the EBV has a latency phase where it infects epithelial cells, enters the circulating B lymphocyte, and persists for the life in a latent state (3). According to epidemiological studies, the EBV is estimated to be positive in more than 90% of the world’s populations (4). Typically, the primary infection is asymptomatic and occurs during childhood. However, the infection could lead to IM if it occurs in adults (5). In addition, this virus has been linked to a wide range of malignancies, such posttransplant lymphoproliferative diseases (PTLDs), nasopharyngeal carcinoma (NPC), Hodgkin’s lymphoma, and gastric carcinoma (MS) (4–8).

The oral route is the primary route of the EBV transmission. However, it has been reported that organ transplantation and blood transfusion can lead to EBV spread (9–11). Through the screening for numerous infectious pathogens, blood banking services spend intense efforts and follow strict precautions to minimize the risk of EBV transmission in transfusion. Nonetheless, concerns regarding the transmission of untested pathogens, such as HEV (12), CMV (13), and EBV (14), are still present. Indeed, blood banks rely on leukoreduction to minimize the number of EBV genome and confirm the safety of blood products. However, it was found that leukoreduction does not eliminate the risk of EBV transmission since the virus can still be detected in leukoreduced blood products (14). Therefore, blood products are considered still potentially dangerous for recipients of blood transfusion, in particular, high-risk individual including organ transplanted and immunocompromised patients (14, 15). However, most EBV studies focus on serological assays (14, 16–19), and limited number of studies have investigated the EBV viremia in healthy blood donors (20–23).

There are two main EBV genotypes, type 1 and type 2, or A and B, respectively, distinguished by the differences in EBNA-2 gene, since the divergence in EBNA-2 reveals only 54% homology between the two types (24). EBV types 1 and 2 can further be subdivided into different virus strains (25). Most of the investigations concerning the genetic variability of EBV strains were based on studying the LMP-1 oncogene since it has a greater degree of polymorphism than most of the others EBV genes (26). Variants in LMP-1 were classified into 7 main groups: B95-8, Alaskan, China 1, China 2, Med+, Med−, and NC (4, 6, 27). However, new LMP-1 strains were reported from different origins such as the Southeastern Asia 1 (SEA1), and Southeastern Asia 2 (SEA2) reported in Thailand (28, 29). Interestingly, it was found that multiple EBV variants could be detected within one individual (25). Moreover, some LMP-1 variants were correlated with cancer progression such as CAO strain, which was isolated from NPC patient is China and has shown to carry atypical 10 amino acid deletion resulted in increased transforming ability (27).

This paper provides insights about EBV in healthy blood donors by reviewing recent reports about the virus epidemiology, serology, and detection, in addition to the genetic variability of LMP-1 oncogene.

## EBV Structure and Genome

The EBV virion structure is similar to other herpesviruses. It consists of a toroid-shaped protein core wrapped with the viral DNA inside an icosahedral capsid of 162 capsomers, a viral tegument containing a protein that lines the space between the nucleocapsid, and the outer envelope, with different glycoprotein spikes inserted into the viral envelop (6, 30).

The EBV genome is composed of a linear, double-stranded DNA with a relatively large genome size of ~ 172 kilobase pairs (kbp) that encodes for more than 85 genes (5, 6). In order to have the highest coding capacity, the viral genome is divided into short and long unique sequence domains, which are formed by a series of around 540-bp terminal direct repeats and around 3.1-kbp large internal repeats (31, 32). These repetitions serve as an indicator to determine whether the source of EBV in the infected cells comes from the same progenitor cell (6). The nomenclature of the EBV open reading frames was established according to a BamHI-restriction fragments map, where the found fragments were ordered in descending order from A to Z based on their sizes. The fragments were also divided into latent or lyric genes (6, 30).

Most of the proteins encoded by the EBV genome are involved in the nucleotides metabolism, to maintain the replication of the viral DNA, and to build the structural compartments of the virus such as the nucleocapsid, tegument proteins, and the envelope (31). Additionally, the EBV genome consists of several latent genes that are non-translated during the lytic phase, along with a number of latency associated RNA genes that are expressed during latency (6, 31). During a latent EBV infection, the viral genome persists for life-long in multiple circular episomes inside the infected cell nucleus. During the cell division, in order to maintain this episome like plasmids, two components are needed: a cis-acting DNA segment (oriP), and a trans-acting nuclear protein (33). In latency, only a few viral genes are expressed, which includes the six EBV nuclear proteins: EBNA-1, EBNA-2, EBNA-3A, EBNA-3B, EBNA-3C, EBNA-LP, in addition to three latent membrane proteins: LMP-1, LMP-2A, LMP-2B (5). Furthermore, although the EBV DNA usually persists in the form of episome, it was found that it can integrate into the cell chromosomal DNA, and persist as integrated DNA as well (34).

## EBV Genotypes and Strain Variation

It has long been known that there are two different EBV genotypes: Type 1 and Type 2, also known as Type A and B, respectively (32). These two genotypes were distinguished based on the differences in the EBNA-2 gene since the EBNA-2 clearly classifies Type 1 and Type 2, where the rest of the EBV genes differ only by less than 5% in their sequence (4, 6). The EBNA-3 gene family also shows variations between the EBV genotypes, but with less degree of sequence difference than the EBNA-2 gene (4). The divergence in EBNA-2 reveals only 54% of homology between the two types, facilitating the distinction between each EBV type (24). Interestingly, it was found that the EBV types noticeably differ in their transformation abilities. For instance, the EBV Type 1 transforms the B lymphocytes more willingly than Type 2 *in vitro*, and when a recombinant Type 2 virus acquired the Type 1 EBNA-2A gene, it gained the transforming ability of Type 1 virus (35).

Epstein–Barr virus Type 1 and 2 can further be subtyped into different EBV strains (25). The genetic variability between the different EBV strains is thought to occur due to the nature of the EBV life cycle within the lymphocytes. For instance, when the EBV infected lymphocyte passes through the germinal center of the lymph node, which is considered a highly mutagenic environment, and thus a location were an increased rate of mutations could occur (36). Consequently, the EBV can induce errors during replication and creates more genetic variability between individuals (36). There are many studies in the literature focusing on investigating the genetic variability of the EBV strains trying to correlate this variability to the geographic areas and the disease outcomes (5). In these studies, genes which were identified to have an important role in the EBV viral life cycle were sequenced, such as BZLF1, EBNA-1, EBNA-2, EBNA-3A, -3B, and -3C, LMP-1, and LMP-2a (28, 37–40).

Interestingly, among the proteins involved in the EBV viral life cycle, LMP-1 is the only protein with oncogenic properties as indicated by its ability to transform rodent fibroblasts and establish tumor cells (41–43). Indeed, a recombinant virus lacking LMP-1 was reported unable to immortalize resting B lymphocytes (44). Many reports indicated that LMP-1 is not only essential for the outgrowth of lymphoblastic cells but also for the survival and proliferation of these cells (45). The oncogenic ability of LMP-1 can be attributed to its effect on a plethora of functional activities including DNA synthesis, suppression of cell senescence, production of cytokines (IL-6, -8, and -10), upregulation of anti-apoptotic proteins (Bcl-2, Mcl-1, Bfl-1, A20, and cIAPs) and cell surface markers (CD23, CD40 ICAM1, LAF1 and LFA3), and epithelial growth factor receptor (41, 46, 47). Furthermore, it has also been shown that LMP-1 is able to induce the activation and secretion of different matrix metalloproteinases suggesting an important role for this oncoprotein in both the angiogenic and metastatic process during the onset and development of EBV-associated tumors (48). Noteworthy is the observation that LMP-1 expression, and in turn all the biological and function effects related to it, can be induced by circulating’s cytokines, a phenomenon that may explain the heterogeneous expression of this viral oncogene both in normal and malignant cells (49).

## The LMP-1 Oncogene Variation

**Figure 1:**
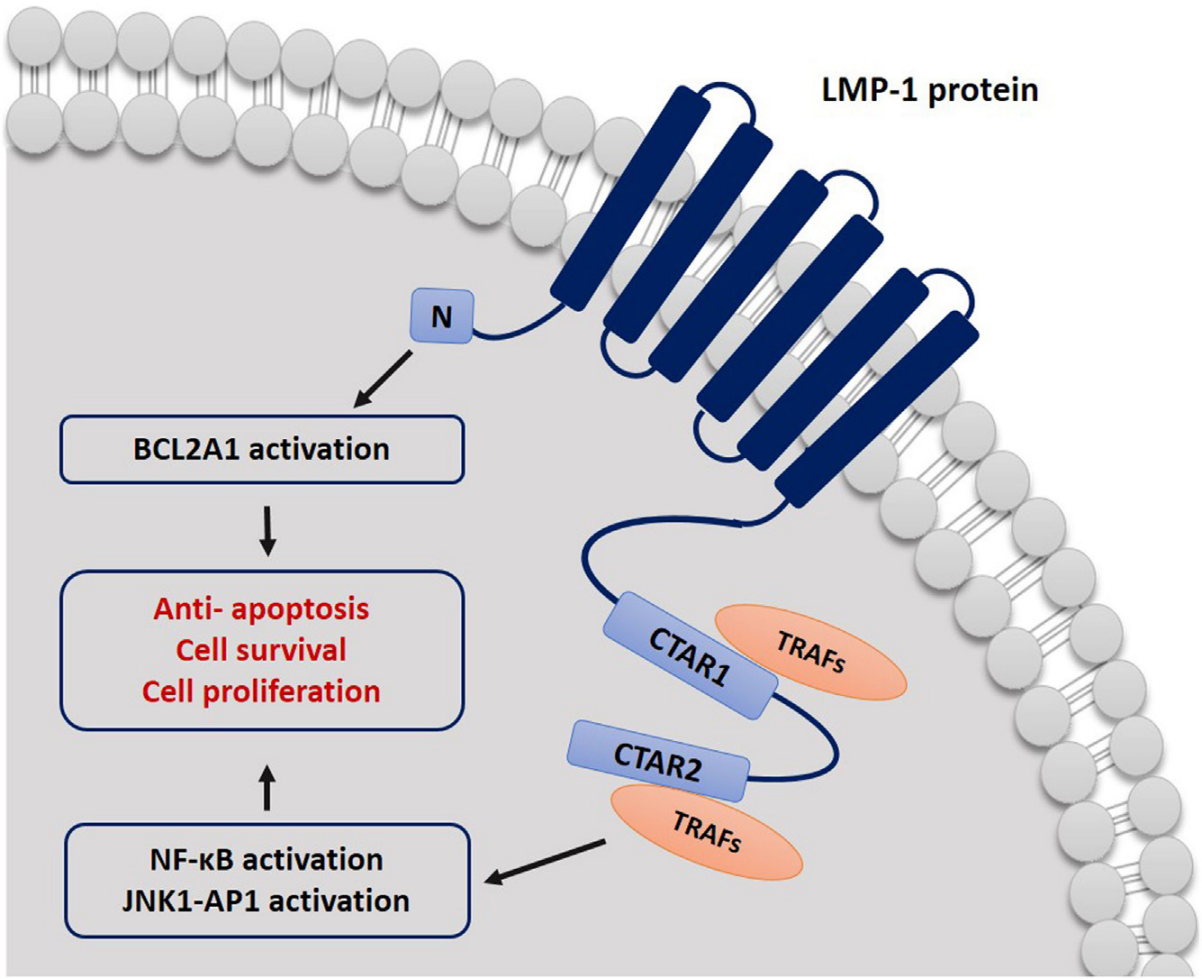
Schematic representation describing the mechanism by which LMP-1 protein affects cell signal transduction. CTAR1 and CTAR2 bind to TRAF proteins and activate NF-κB and JNK–AP-1 pathways. LMP-1 can block cell apoptosis signals by activating BCL2A1.

Most of the recent investigations on the EBV strain variation were based on studying the LMP-1 oncogene sequence, because it has shown to have a greater degree of polymorphism than most of EBV genes between different strains (4). LMP-1 is a 356-amino acid protein, which consists of a short cytoplasmic N-terminus, six membrane-spanning domains, and a long cytoplasmic C-terminal domain (26). The cellular signaling pathways targeted by LMP-1 share functional characteristics with members of the tumor necrosis factor (TNF) receptor superfamily. Molecular studies have revealed that the C-terminal domains (CTAR1 and CTAR2) of LMP-1 play an important role in signal transduction through mimicking the CD40-mediated signaling (50). The LMP-1 protein binds the tumor necrosis factor receptor-associated factor (TRAF) proteins and the TNF receptor-associated death domain protein (TRADD) activating several intracellular pathways including NF-κB, the mitogen-activated protein kinases JNK and p38, the small GTPase Cdc42, and the JAK/AP-1/STAT cascades. Activation of these intracellular signaling cascades enhances cell survival and proliferation and may account for many of the cellular changes observed in response to LMP-1 (50–52). Moreover, the LPM1 protein works as homologous to the TNF-receptor family in the B lymphocytes and epithelial cells (6). Therefore, when the LMP-1 protein is mutated, sequence variation can affect cell process directly as it interferes with major cellular signaling pathways (4). It is well known now that LMP-1 is essential in the transformation of B lymphocytes into a lymphoblastoid cell line, and it has the ability to block apoptosis by upregulating different anti-apoptotic proteins such as A20 and Bcl-2 and inhibiting the p53-mediated apoptosis (6) (Figure 1).

Based on the LMP-1 sequence variation, the EBV strains were classified into seven main groups/variants relative to the wild-type strain B95-8. The nomenclature of these variants reflects their geographic origin or the location from where they were found: Alaskan (Ala), China (Ch1) and (Ch2), Mediterranean (Med+) and (Med−), and North Carolina (NC) (4, 27). However, new strains were reported from different origins. In Thailand, two other new variants were found which were named: SEA1, and SEA2. The Chinese del-LMP-1 (CAO) isoform variant was also isolated from NPC patients (11, 12).

Multiple EBV variants can be detected within one individual, as a patient can be infected with more than one type (25). There is evidence of specific multiple LMP-1 variants found in people infected with mononucleosis, EBV-associated malignancies such as Hodgkin Lymphoma and NPC, as well as in *human immunodeficiency virus* (HIV) patients (4). Interest in LMP-1 variants has increased when findings correlating LMP-1 variants with specific cancers were reported. For instance, a variant with 30-bp deletion was frequently detected in NPC patients, and this variant showed higher transforming activity than the typical LMP-1 (53). Furthermore, a 69-bp deletion variant has also been reported in Burkitt’s lymphoma and at a lesser rate also in NPC. Additionally, the 69 bp deletions were also correlated with a decreased activation of the AP-1 transcription factor (4, 54). Several reports also investigated the presence of LMP-1 variants among healthy carriers (20, 25, 55). A recent study compared the prevalence of EBV genotypes and del-LMP-1 among Polish, Taiwanese and Arabic healthy individuals revealed that 62.5% Taiwanese and 55.6% Polish had a 30-bp deletion in the LMP-1 gene. However, the study reported that this deletion was not present in the Arabs population (20). Another study investigated the frequency of the 30-bp deletion in EBV healthy carriers from Argentina and found that it was present in 28% of these healthy people (55). In our study investigating the molecular variability of LMP-1 gene in healthy donors, the 30-bp deletion was observed in 30.6% of study subjects (23).

## EBV Viral Life Cycle and Activation

**Figure 2:**
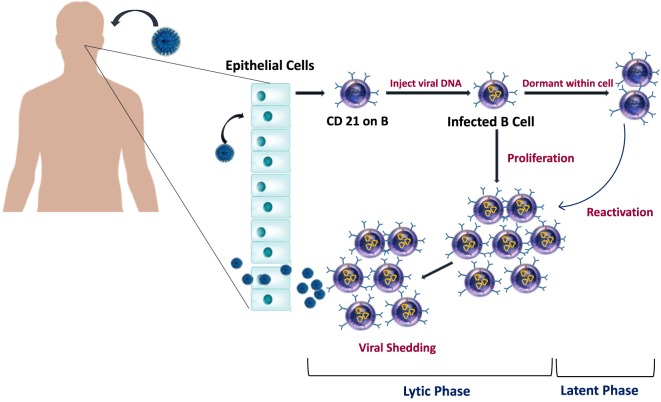
Epstein–Barr virus (EBV) life cycle in healthy carriers. The infection begins when EBV infect epithelial cells and naïve B cells of the oral cavity. EBV genome will be transported to the nucleus of B cell where it will replicate and results in the proliferation of B cells. Latency occurs when EBV downregulate most of its protein-encoding genes. Later, as cells recirculate between peripheral and oral compartments, resting B cells will be reactivated and cause viral shedding.

The EBV usually spreads through the saliva, then it enters the epithelium of the tonsils and starts the lytic phase of infection that involves virus replication (6) (Figure 2). Infected naive B lymphocytes become activated lymphoblasts and migrate to the lymph node follicle to initiate a reaction in the germinal center of the follicle using the “latency III” program, where all latent growth proteins are expressed and adversely regulate the EBV growth. Among the virus proteins expressed during this phase are the EBV nuclear antigens (EBNA-1, -2, -3, -3A, -3B, -3C, and -LP), and latent membrane proteins [LMPs (LMP-1, -2A, and -2B)] (6, 56). Type II latency program then is initiated in which only EBNA-1, the EBERs, the BARTs, LMP-1, and LMP-2A are expressed (56), and survival signals will be provided to cells to move out of the germinal center as memory B lymphocytes (6). The “Latency 0” phase begins in the memory B lymphocytes, and it is characterized by arrest all the viral proteins expression (6). If only the EBNA-1 gene is expressed when these memory B lymphocytes divide, then the phase is called “latency type I” (33, 57). The infected memory B lymphocytes can also migrate back to the tonsils, where they can induce more viral replication and spreading and thus infect other B lymphocytes as well (3). In the primary infection, T lymphocytes are responsible for eliminating the newly infected cells and controlling the infection. However, during latency, the EBV is hidden from the immune system as it remains silent in the resting memory B lymphocytes without expressing any viral protein (6, 58).

Viral reactivation can occasionally happen in latently infected memory B lymphocytes and leads to a new viral cycle, where it replicates, infects new cells, and sheds in the saliva (56). Under healthy conditions, immunocompetent individuals can have EBV reactivation with no specific symptoms due to the infection control by the cytotoxic T lymphocytes (59). However, EBV reactivation can be life threatening in patients under immunosuppression and can lead to severe EBV-related pathologies, such as posttransplant lymphoproliferative disorders (PTLDs) (59). There are several described causes of EBV reactivation, including the presence of foreign antigen that leads to memory B lymphocytes division, which in turn can induce viral reactivation and replication (60), meaning that any new infection can lead to EBV reactivation (6). For instance, malaria infection has been linked to EBV reactivation, as *P. falciparum* antigens can directly trigger EBV reactivation and, therefore, can increase the risk of developing Burkitt’s lymphoma in malaria endemic areas (61). The cystein-rich inter-domain region 1α in the *P. falciparum* membrane protein 1, was found to activate the memory B lymphocytes where the EBV resides (61). Other causes of virus reactivation are immunodeficiency and immunosuppression, which are due to altered immune system. In this case, uncontrolled reactivation of EBV may occur and can lead to various lymphoproliferative diseases (59). Other factors, such as inflammation and chemical agents or drugs, have also been linked to EBV reactivation from latently infected cells (6).

Many studies have been conducted to investigate the EBV host cell interactions and the latency associated with the EBV infection in different cell types and various medical conditions (62). In healthy hosts, B lymphocytes and epithelial cells are the cellular targets for EBV primary infection. However, the EBV can infect a wide range of non-B lymphocytes, and it critically affects the development and pathogenesis of EBV-related diseases (63). Early studies reported the presence and replication of EBV viral particles in the oropharyngeal epithelial cells of patients with acute IM (64, 65), and in epithelial cells of HIV patients suffering from oral hairy leukoplakia (66). More recent studies showed that the tonsil epithelium of asymptomatic patients has the ability to carry EBV infection, which is a part of the viral life cycle (67). Furthermore, the EBV can also infect T lymphocytes, plasma cells, NK cells, monocytes, follicular dendritic cells squamous, myoepithelial and glandular epithelial cells, and smooth muscle cells (68–72).

Despite the wide range of suspected cell types involved in the EBV infection, it appears that B lymphocytes have a critical role in the viral life cycle, as agammaglobulinemia patients, who have a genetic mutation that leads to the absence of mature B lymphocytes, are not affected by EBV (73). Primary B lymphocytes can be easily infected with the EBV since B lymphocytes possess a major receptor molecule of the virus called cellular complement receptor type 2 (CR2 or CD21), which binds to the EBV glycoprotein gp350/220 (56). On the other hand, the interaction of EBV with epithelial cells is less understood. It appears that epithelial cells acquire the infection through transfer from EBV-coated B lymphocytes (62). *In vitro* studies showed that a low rate of infection was achieved when epithelial cells were exposed to cell-free virus preparations, while a quantifiable level of infection was reached when epithelial cells were cultured with EBV infected B lymphocytes. This prompts the idea of the importance of B lymphocytes in the infection (74). Moreover, EBV might enter the epithelium through the surface of resting B lymphocyte. B lymphocyte can act as a shuttle, to transfer the EBV infection to CD21 negative epithelial cells after the EBV binds to its surface (74). However, it is still in doubt whether B lymphocytes or epithelial cells are the primary targets of EBV spread (31).

## EBV Transmission and Seroprevalence

The main route of the EBV transmission is the oral route, as it is generally transmitted through the saliva that contains infected epithelial cells (75). Also, it can spread through the blood, by means of blood transfusion and organ transplantations (1, 9, 11, 14). Infected epithelial cells can also be found in the uterine cervix or in the semen, suggesting the possibility of EBV spread through sexual contact (75). Kissing, sharing personal objectives such as toothbrushes, eating utensils, or sharing food and drinks with an infected individual can all lead to EBV spread (1).

In healthy individuals, the EBV is highly prevalent, as it affects more than 90% of individuals worldwide (17). The age of primary infection was found to vary according to socioeconomic factors that are reflected by crowdedness and low sanitation (6). The EBV seroconversion occurrence has two patterns. In developed countries with high hygiene standards, the EBV seroconversion peaks in children between 2 and 4 years and also in 14 and 18 years, and it increases with age, ranging from 0 to 70% at childhood and reaching to more 90% in adulthood (14). Contrary, in countries with poor hygiene standards, the EBV infection is usually acquired in early childhood, and almost all children in those developing countries are seropositive by the age of 6 years (75).

## Distribution of EBV Genotypes

Epstein–Barr virus types occur worldwide, but they differ in their geographic distribution. For instance, Type 1 is prevalent in population from Europe, America, China, and South Asia, while Type 2 is less prevalent in these populations and is more observed in African and Papua New Guinean populations, where it shares an equal distribution with Type 1 (6, 76). Immunocompromised patients are more susceptible to acquire both types (6). However, healthy individuals as well can have mixed infection with both Type 1 and 2 (25). In a recent study conducted on healthy blood donors in Qatar (23), we have reported a predominance of the genotype 1 (72.5%) as compared to the genotype 2 (3.5%), and mixed infection with both genotypes was detected in 4% of the samples. Nonetheless, it is still not known how many EBV variants can be found in one individual, and whether the immune system of a previously infected individual provides protection against new multiple variants (36).

## EBV in Blood Donation and Organ Transplantation

It has long been known that blood transfusions and organ transplantations can be routes for EBV transmission, as reported in 1969 by Gerber et al. (9). In this study, it was shown that patients receiving donor blood during an open heart operation acquired the EBV infection, indicating the possibility of EBV transmission by blood transfusion. Furthermore, early studies revealed that the EBV transmission could also occur through organ transplantation, where patients developed IM after transplantation (10, 11). Reports showed that a healthy EBV seropositive individual carries around 0.1–50 EBV infected B lymphocytes per 1,000,000 peripheral blood mononuclear cells. Therefore, it is possible that EBV can be transmitted through the white blood cells of the blood (14, 77).

The majority of the epidemiological studies on viral infections including EBV were based on serological assays rather than on the detection of the EBV viremia (14, 16–19). Nonetheless, measuring the amount of circulating viral load can better reflect the infection status (78–80). A limited number of studies have investigated the EBV viremia in healthy individuals (20–23). A study performed in us showed that 72 of a 100 randomly selected blood donors had a detectable EBV DNA, suggesting that the potential for transfusion-mediated transmission of EBV is high (22). In Japan, randomly selected blood donors were tested for the presence of EBV DNA and the results showed that the EBV DNA was detected in 39.5% of the donors (81). Another recent study in Burkina Faso showed a lower level of EBV rate among blood donors, as it was detected in only 5.1% of the donors (82). Previous studies from Middle Eastern countries, including Saudi Arabia (83), Kuwait (84), the UAE (85), Egypt (86), Jordan (87), and Syria (88), have studied the association of EBV and multiple diseases such as Hodgkin’s lymphoma (reported prevalence of 28–87%), but EBV serological and molecular prevalence among healthy individuals was not investigated. We have recently studied the rate of EBV infection among 673 healthy blood donors from different nationalities in Qatar (23), we reported a seroprevalence of 97.9%, and a viremia rate of 52.6%, with a viral load ranged between 0.915 and 2,585.5 copies/ml of blood. Both EBV seroprevalence and viremia rates increased significantly with age (23).

The EBV has been linked to the development of posttransplant lymphoproliferative disorders (PTLDs) which is a group of heterogeneous diseases that develop in immunocompromised patients after receiving a solid organ or hematopoietic stem cell transplant (89). The incidence of lymphoproliferative disorders increases with solid organ and bone marrow transplantations (90, 91). PTLDs develop as a result of immunosuppression, and they vary from benign slow polyclonal proliferations to overtly malignant monoclonal proliferations of lymphocytes and plasma cells (89, 92). PTLD was first reported in 1968 in two renal transplantation recipients, and it was linked to the immunosuppressive therapy that was administered to the patients (93). Mortality from PTLDs is high with no progress in the outcomes over the years (94). The World Health Organization classifies PTLDs to (i) early lesions of polyclonal or oligoclonal lymphoid proliferations that are mainly derived from EBV infection and (ii) late monoclonal lymphoproliferative diseases that are not necessarily associated with EBV, including polymorphic and monomorphic PTLDs, which also can be subdivided into Burkitt’s lymphoma, Burkitt’s-like lymphoma, diffuse large B-cell lymphoma, and Classical Hodgkin Lymphoma (95). It has been known that oncogenic herpesviruses like EBV and HHV-8 are involved in the development and pathogenesis of PTLDs because these viruses have the ability to infect and transform B lymphocytes directly (67). Indeed, EBV was found to be present in up to two thirds of the PTLD cases (89). A higher risk to develop PLTDs is found in EBV negative than in EBV-positive recipients regardless the status of the donor, but the highest risk is when the recipient is EBV negative, and the donor is EBV positive (96).

Efforts to prevent the transmission of the EBV from EBV-positive donors rely on the process of leukoreduction, which was introduced in 1999, and aimed to remove the white blood cells from various blood products (96). In a study of leukoreduction efficacy, Qu et al. concluded that EBV PCR negative blood products after leukoreduction, are expected to have a very low probability of transmissible EBV, and thus the risk is highly reduced (97). However, a recent study showed that the EBV was detected in one platelets bag after leukoreduction (15). The above finding indicates that the leukoreduction does not rule out the possibility of EBV transmission, and leukoreduced blood products can harbor the EBV. Consequently, there might be a potential risk in immunocompromised patients who are more vulnerable to EBV infection, and patients who receive large volumes of blood (15).

## Detection of EBV

The clinical presentation of the EBV infection usually overlaps with other acute viral syndromes caused by other viruses such as CMV and hepatitis viruses, which can lead to similar symptoms (98). The above aspect emphasizes the importance of having reliable laboratory diagnostic tools that help in the differential diagnosis. Diagnostic schemes of EBV differ according to the patient’s immune condition because the importance and urgency of therapeutic intervention differ between immunocompromised and immunocompetent individuals. A wide range of assays was utilized in the diagnosis of EBS. This includes the use of nonspecific tests such as heterophile antibodies detection (mono spot test), EBV specific serological assays such as ELISA, EIA, IFA, chemoluminescence, immunoblot, and IgG avidity, and molecular assays for nuclear acid detection (99). Other diagnostic tools also have been used in the detection of EBV-associated tumors such as immunhistochemistry and immunocytology (99, 100).

## Serological Testing

**Figure 3:**
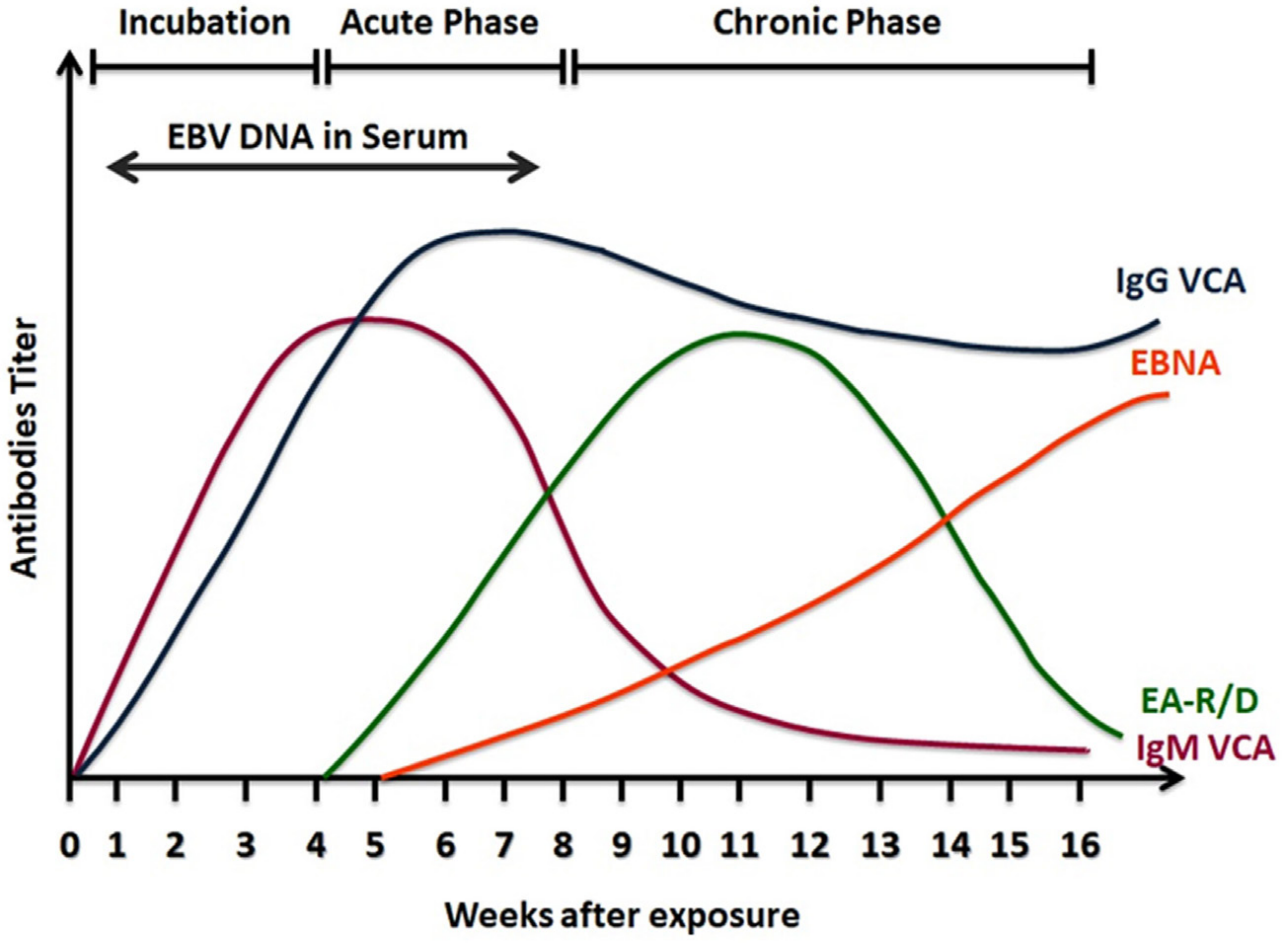
A scheme of serological response to Epstein–Barr virus (EBV) infection. Viral capsid antigens (VCA)-IgM is detected in the active phase of infection and then declines in convalescence. VCA-IgG increases at the same time of VCA-IgM, but it remains positive for life indicating past infection. Epstein Barr nuclear antigens (EBNA) antibodies are detectable late in the phase of infection and also remain positive. Early antigens (EA) antibodies to the class R or D increase in the acute phase of infection and decline after convalescence.

Serological testing is based on the detection of EBV antibodies in the patient’s serum. Although the serology for EBV diagnosis shows a high degree of variability, it is still preferred and commonly used compared other tests, as it provides reasonable criteria to identify the patient’s infection status (99). The EBV genome codes for different structural and nonstructural genes, some of these genes are used in the serological diagnosis, as the humoral response produces antibodies against the product of these genes. Among the genes used in the test are genes that codes for the viral capsid antigens (VCAs), the early antigens (EAs), as well as the genes that code for Epstein–Barr nuclear antigens (EBNAs) (98, 99). The heterophile test is also one of the commonly used tests to help in the clinical diagnosis. This test is based on heterophile antibodies detection which are antibodies that agglutinate erythrocytes from animal sources and is mainly linked to mononucleosis caused by the EBV infection or infrequently by other diseases (101). This test is easy to perform, inexpensive, and commercially available, but it lacks specificity, as false-positive results were reported in other conditions such as in autoimmune diseases and cancers which were found unrelated to the EBV infection (102). Moreover, this assay shows low sensitivity with high false negative results when used for children younger than the age 2 years old, as they might lack specific EBV antibodies (101, 103). In immunocompetent individuals, usually at least three serological parameters are needed to detect EBV antibodies: VCA-IgG, VCA-IgM, and EBNA-1 IgG (104). Detection of IgG antibodies to EBV EA can also be done and helps in the differentiation of the EBV diseases status (99, 101) (Figure 3).

The VCA is a complex of seven structural proteins and glycoproteins, and it is synthesized in the lytic cycle of the EBV replication (105). The serodiagnosis of VCA is based on the detection of antibodies against the two recombinant VCAs: the N- terminal of full length p23 and the carboxy half of p18 (106). These two proteins were joined *in vitro* by autologous gene fusion in 1999, establishing the bases for developing novel EBV ELISAs (106, 107). VCA antibodies detection involves the two immunoglobulin classes IgG and IgM. The humoral response to the VCA complex is typically found early at the onset of clinical symptoms (102). In a study investigating EBV status in college students, VCA-IgM was detected by enzyme immunoassay 8 days earlier than the onset of the symptoms (102). VCA-IgM is produced transitionally and used as an indicator of recent primary infection. Indeed, VCA-IgM is no more detected after convalescence, and generally it does not occur another time in life (99). Although VCA-IgM appears early and helps in the diagnosis of acute EBV infection, some limitations that interfere with the accurate interpretation of the results are present. For example, some children and adults might have negative VCA-IgM in primary acute infection (101), and EBV-IgM cross-reactivity with other antigenically related infections especially CMV (108, 109). VCA-IgG is found in acute, convalescence, or past infections, as it starts to appear at the same time as VCA-IgM (99, 101, 106). Antibodies against the p18 components develop after p23 antibodies and then persist for life as an indication of EBV exposure (102). Measuring VCA-IgG antibodies are found to be a best single test to indicate a previous EBV infection as all patients with IM produce IgG antibodies to VCA (110).

The EBV nuclear antigen (EBNA) is composed of six proteins (EBNA-1, -3, -3A, 3B, 3 C and LP) (3). The EBNA-1 protein is expressed in all EBV infected cells, and IgG against this protein is a late marker of EBV infection (110). EBNA-1 IgG antibodies appear late, 3 to 6 months after the time of disease, then they decline but continue to be present in a detectable level for life Thus, detection of EBNA-1 antibodies indicates past or recovering EBV infection (6, 110). However, VCA-IgG indicates past infection more accurately than EBNA-IgG because EBNA-IgG is never developed in around 5–10% of EBV infected healthy individuals, and this percentage is higher in immunocompromised patients (102, 111). Usually, antibodies against EBNA are tested by standard immunofluorescent assays and enzyme immunoassays. However, EBNA enzyme immunoassays may give false-positive results (1, 101, 104). The IgM class of EBNA-1 is not usually measured, but when detected, it indicates a recent primary infection, however, it may persist for several months after the primary infection, and it can reappear again in the reactivation process (101). The EBNA-1 IgM has cross-reactivity with other viruses such as CMV and Parvovirus B19, and it may show false negative results (112, 113).

The EA is a complex of nonstructural proteins that are expressed by EBV infected cells in the lytic phase. EA is composed of two components: diffused EA-D and restricted EA-R (114). IgG antibodies against EA are detected transiently in up to 3 months or more during infection mononucleosis (111). Usually the humoral response is against the D component; however, children undergoing silent EBV seroconversion might also produce antibodies to the R component (101, 115). High levels of EA-R antibodies have been detected in Burkitt’s lymphoma (101), and can also be indicative of reactivation of a latent EBV infection (116). In contrast, high titers of EA-D were found to be produced in NPC patients (117). Hence, detection of only EA antibodies cannot serve as an ultimate diagnosis to identify the EBV condition, because high titers are found in different diseases, and in healthy individuals as well (118). Usually, EA antibodies appear in the acute phase and then declines to undetectable levels. However, studies showed that only 60–85% of acute infection patients have EA positive results (99, 102) and 20–30% of healthy individuals with past EBV infection have detectable levels of EA antibodies (99). Because of abovementioned reasons, the diagnostic value of these antibodies is still debatable (101). Nevertheless, combining EA antibodies testing with other diagnostic tools can be useful in the diagnosis.

In general, the EBV infection in immunocompetent patients is detected and classified using the previously mentioned antibodies in patients’ sera. However, when results are uninterpretable or cannot clearly distinguish the stage of infection, other assays can be done to confirm the suspected diagnosis, such as western blot, immunoblot, and more commonly, the IgG avidity testing (18, 102).

## IgG Avidity Assay

Due to the high variability and cross-reactivity in EBV serological responses, mainly with the VCA antibodies, more parameters are occasionally needed to confirm the infection condition. IgG avidity assay is usually employed in combination with other serologic markers. This method is based on the principle that during the acute phase of infection, the binding strength of EBV IgG antibodies to their target antigens is not as high as the antibodies binding strength after finishing the acute infection, as the antibodies undergo maturation (101, 119). Treating low avidity IgG antibodies with urea or chaotropic reagent leads to antibodies disassociation. Consequently, the difference in the antibodies amount before and after urea treatment is evaluated to determine the avidity strength which in turn represents the stage of infection and distinguish acute from past infection (102, 120). This method was found to be a reliable tool in EBV primary infection confirmation in patients with undetectable VCA-IgM, as well in the differential diagnosis (18, 120).

## Molecular Assays

Various molecular techniques have been developed and applied to detect EBV DNA and to quantify the viral load (68, 121–123). *In situ* hybridization, RNA and protein based assays, detection of EBV DNA in blood samples by quantitative real-time PCR (qRT-PCR), Southern blotting and Dot blotting have all been used in the diagnosis and monitoring of primary EBV infection, reactivation, and in EBV-related diseases (68, 124). These methods aid in the diagnosis, but due to the lack of standardization, the difference in sensitivity and specificity from the laboratory to laboratory should always be considered (101).

A growing body of evidence indicates the importance of using qRT-PCR as a sensitive and reliable method and a complementary tool to other serologic markers, in particular, for diagnosis of EBV acute infection and EBV silent reactivation (59, 103, 125, 126). More importantly, this method is very crucial and widely used in monitoring the immune status of immunocompromised patients as well as in patients at risk of developing EBV-related diseases (127–129). However, the threshold value in which medical intervention is required, the units of measurement, and the best specimen to be used for DNA testing are still questionable and not standardized (101). Moreover, there is still no consensus on the ideal method for performing qRT-PCR in case of EBV detection and quantification, and this increases the variability between laboratories and between studies (68). Different detection methods are available commercially. Some commercial primers and probes target include LMP-2 gene, BHRF-1 (a transmembrane protein), BKRF1 (EBNA-1 gene), BNRF1 (a major tegument protein), BXLF1 (thymidine kinase), BZLF1 (ZEBRA), or BALF5 (viral DNA polymerase) (68, 101) Table 1 summarizes the most commonly used primers in the detection of EBV DNA. The unit of measurement also varies; it can be reported as copies per DNA concentration as copies per microgram of DNA, or copies per milliliter, copies per 100,000 white blood cells, and copies per positive cell (68, 101, 130). Samples that used in qRT-PCR assays are various, including serum, whole blood, tissue biopsy, and peripheral blood mononuclear cells (PMNCs). Although there is still debate concerning these issues, in general, the choice of the specimen to be used is based on the patient’s condition and the stage of the disease. Studies on the EBV life cycle showed that production of EBV virions during the acute phase of infection and the degradation of EBV DNA by apoptotic cells, both lead to the spread of virions and free or degraded EBV in the peripheral blood of the patient, and therefore, this allows for EBV DNA detection in patients’ peripheral blood (124, 131, 132). In the latent phase of the infection, transformed B lymphocytes also travel to the blood (101). Consequently, EBV DNA in acute infection can be detected in the serum or in the unfractionated blood, as well as in the PMNCs. Table 2 describes the molecular prevalence of EBV DNA using different sample types.

**Table 1:** Primers used for detection Epstein–Barr virus (EBV) DNA.

Gene/region	Method	Primers	Reference
EBNA-1 gene	Nested PCR	-Outer primers: Forward primer: 5′-GTA GAA GGC CAT TTT TCC AC-3′ Reverse primer: 5′-CTC CAT CGT CAA AGC TGC A-3′ -Inner primers: Forward primer: 5′-AGA TGA CCC AGG AGA AGG CCC AAG C-3′ Reverse primer: 5′-CAA AGG GGA GAC GAC TCA ATG GTG T-5′	(133)
	
	Real-time PCR	Forward: 5′-TCATCATCATCCGGGTCTCC-3′ Reverse: 5′-CCTACAGGGTGGAAAAATGGC-3′ Probe: 5-(FAM)-CGCAGGCCCCCTCCAGGTAGAA(TAMRA)-3′	(134)
		
		Forward: 5′-GACTGTGTGCAGCTTTGACGAT-3′ Reverse: 5′-CGGCAGCCCCTTCCA-3′ Probe: 5′-(FAM)-TAGATTTGCCTCCCTGGTTTCCACCTATG-(TAMRA)-3′	(20, 134–136)

BamH1-K	Real-time PCR	Forward primer: 5′-CCG GTG TGT TCG TAT ATG GAG-3′ Reverse primer: 5′-GGG AGA CGA CTC AAT GGT GTA-3′ Probe: 5′-TGC CCT TGC TAT TCC ACA ATG TCG TCT T-3′ (SEB).	(137)

EBNA-2 Gene	Nested PCR	-Outer primers: Forward primer: 5′-AGGGATGCCTGGACACAAGA-3′ Reverse primer: 5′-TGGTGCTGCTGGTGGTGGCAAT-3′ -Inner primers: *EBV type 1* Forward primer: 5′-TCTTGATAGGGATCCGCTAGGATA-3′ Reverse primer: 5′-ACCGTGGTTCTGGACTATCT-GGATC-3′ *EBV type 2* Forward primer: 5′-CATGGTAGCCTTAGGACATA-3′ Reverse primer: 5′-AGACTTAGTTGATGCCCTAG-3′	(23)
		
		-Outer primers: Forward primer: 5′-TTT CAC CAA TAC ATG ACC C-3′ Reverse primer: 5′-TGG CAA AGT GCT GAG AGC AA-3′ -Inner primers: Forward primer: 5′-CAA TAC ATG AAC CRG AGT CC-3′ Reverse primer: 5′-AAG TGC TGA GAG CAA GGC MC-3′	(20)
		
		-Outer primers: Forward primer: 5′-TGGAAACCCGTCACTCTC-3′ Reverse primer: 5′-TAATGGCATAGGTGGAATG-3′ -Inner primers: Forward primer: 5′-AGGGATGCCTGGACACAAGA-3′ Reverse primer: type 1 EBNA-2:5′-GCCTCGGTTGTGACAGAG-3′ type 2 EBNA-2:5′-TTGAAGAGTATGTCCTAAGG-3′	(138)

EBNA-3C	Conventional PCR	Forward primer: 5′-AGAAGGGGAGCGTGTGTTGT-3′ Reverse primer: 5′-GGCTCGTTTTTGACGTCGGC-3′	(139)

EBNA-5 BamHI-W Fragment	Real-time PCR	Forward primer: 5′-AGGCTTAGTATACATGCTTCTTGCTTT-3′ Reverse primer: 5′-CCCTGGCTGATGCAACTTG-3′ Probe: 5′-GCAGCCTAATCCCACCCAGACTAGCC-3′	(140)
		
		Forward primer: 5′-CCCAACACTCCACCACACC-3′ Reverse primer: 5′-TCTTAGGAGCTGTCCGAGGG-3 Probe: 5′-(FAM)CACACACTACACACACCCACCCGTCTC-3′	(139, 141)
	
	Conventional PCR	Forward primer:5′-CCAGACAGCAGCCAATTGTC-3′ Reverse primer: 5′-TAGAAGACCCCCTCTTAC-3′	(139)
		Forward primer: 5′-ACC TGC TAC TCT TCG GAA AC-3′ Reverse primer: 5′-TCT GTC ACA ACC TCA CTG TC-3′	(137, 139)

LMP-1 gene	Nested PCR	-Outer primers: Forward primer: 5′-AGTCATAGTAGCTTAGCTGAA-3′ Reverse primer: 5′-CCATGGACAACGACACAGT-3′ - Inner primers: Forward primer: 5′-AGTCATAGTAGCTTAGCTGAA-3′ Reverse primer: 5′-CAGTGATGAACACCACCACG-3′	(23)
	
	Conventional PCR	Forward primer: 5′-AGCGACTCTGCTGGAAATGAT-3′ Reverse primer: 5′-TGATTAGCTAAGGCATTCCCA-3′	(20)

LMP-2 gene	Real-time PCR	Forward primer: 5′-AGCTGTAACTGTGGTTTCCATGA-3′ Reverse primer: 5′-GCCCCCTGGCGAARAG-3′ Probe: 6-FAM-CTGCTGCTACTGGCTTTCGTCCTCTGG-TAMRA	(23)

BALF5 gene	Real-time PCR	Forward primer: 5′-CGGAAGCCCTCTGGACTTC-3′ Reverse primer: 5′-CCCTGTTT ATCCGATGGAATG-3′ Probe: 5′-TGTACACGCACGAGAAATGCGCC-3′	(136)

BamHI-F region	Conventional PCR	Forward primer: 5′-TCC CAC CTG TTA CCA CAT TC-3′ Reverse primer: 5′- GGC AAT GGG ACG TCT TGT AA-3′	(139)

EBER1	Conventional PCR	Forward primer: 5′-TCTGTGGCAGGAGTGGTGGGCCCTGAACAT-3′ Reverse primer: 5′-AGACACCGTCCTCACCACCCGGGACTTGTA-3′	(139)

**Table 2:** Prevalence of Epstein–Barr virus DNA in various samples.

Country	Sample type	Sample size	Seroprevalence (%)	Genotype	Diagnostic assay used	Year	Reference
United States of America	Whole blood	143	42 (29.3)	–	Real-time quantitative polymerase chain reaction	2012	(133)
	Whole blood	92	75 (82)	–	In-house quantitative real-time polymerase chain reaction	2012	(134)
	Plasma	116	15 (13)				
	PMNCs	64	56 (88)				
	Oral wash: cell pellet Supernatant	143	66 (46) 61 (42.6)				
	Whole blood	19	5 (26)	–	Real-time quantitative polymerase chain reaction	2016	(147)
	Whole blood	66	42 (64)	–	Real-time quantitative polymerase chain reaction	2013	(133)
	Whole blood	86	7 (8)	–	Real-time quantitative polymerase chain reaction	2016	(135)

Colombia	Saliva	17	9 (52.9)	–	In-house Real-time polymerase chain reaction	2016	(148)

Brazil	Saliva	100	60 (60)	–	Nested polymerase chain reaction	2018	(149)
	Saliva and fresh tissue samples	17 each	64.7 35.3	–	Nested polymerase chain reaction	2016	(150)
	Scraping samples of the tongue lateral border	53	53 (100)	Type 1,2	Nested polymerase chain reaction	2008	(151)

Australia	Tissue	55	55 (100)	Type 1, 2	DNA sequence analysis	2012	(152)
**Europe**							
Czech Republic	Whole blood	29	19 (66)	–	Real-time quantitative polymerase chain reaction	2011	(153)
	Plasma	29	22 (76)				

Poland	Fresh frozen tumor tissue	78 Oropharyngeal cancer	40 (51.3)	–	Nested polymerase chain reaction	2016	(154)
	Saliva	40 healthy	8 (20)				
	Saliva	56	22 (39.3)	Type 1	Nested polymerase chain reaction	2004	(55)

Sweden	Cervical secretions	305	32 (10.5)		Real-time quantitative polymerase chain reaction		(155)

Germany	Saliva	47	14 (30)	–	Polymerase chain reaction	2017	(156)

Serbia	Tissue	80	37 (46.6)	Type 1	Nested polymerase chain reaction	2016	(147)
**Asia**							
Qatar	PMNCs	673	354 (52.6)	–	Real-time quantitative polymerase chain reaction	2013	(23)

China	PMNCs	859	206 (24)	*–*	Polymerase chain reaction and restricted fragment length polymorphisms (RFLP)	2017	(137)
	Plasma	1,318	69 (5.2)	*–*	Real-time polymerase chain reaction	2013	(141)
	Saliva	20	20 (100)	Type 1,2	Quantitative polymerase chain reaction	2015	(76)
	Paraffin-embedded tissues	209	146 (69.9)	Type 1,2	Quantitative polymerase chain reaction	2014	(157)

India	Serum	40	37 (92.5)	–	Standard phenol chloroform method and then polymerase chain reaction	2016	(158)
**Africa**							
Kenyan	Purified T-cell fractions saliva and breast milk	–	–	Type 2	–	2017	(159)

Egypt	Paraffin-embedded samples of breast tissue	84	32 (38)	–	Nested polymerase chain reaction	2017	(160)

Eritrea	Formalin-fixed paraffin-embedded breast cancer tissue	144	40 (27.77)	–	Polymerase chain reaction	2017	(161)

Sudan	–	150	92 (61.3)	–	Polymerase chain reaction	2015	(162)

The EBV DNA in acutely infected patients can be detected within 2 weeks of the onset of symptoms, and it reaches its peak of detection during this time (101). Then, after the initiation of the immune response, the viral load starts to decrease rapidly to low or even undetectable levels in the plasma or serum (125). After that, the immune response decreases slowly in the cellular portion of blood, where it remains latent in the memory cells for a long time, and thus it can be detected if the sample is PMNCs (68, 142). However, it is important to consider the individual differences in EBV kinetics between patients as the viral load might take up to one year to reach a stable low level in some individuals according to immune status and the patient’s condition (68, 143). Studies showed that healthy individuals carry a stable number of EBV infected cells (81). In a healthy carrier, latently infected memory B lymphocytes harbor the EBV genome, approximately, per 1 million leukocytes, there are 1–50 copies of EBV DNA, while in serum or plasma EBV DNA is almost below the limit of detection (68) for the same individual. Therefore, the ability to detect EBV DNA in serum might serve as a useful indicator for EBV primary infection or reactivation. Patients with active infection or with EBV-related cancers have been found to have a higher viral load in their cell-free blood (68).

Epstein–Barr virus DNA detection in patient’s serum can be useful especially in the early stages of the acute infection, and it can be even more sensitive than serology and IgG avidity testing as previously reported (125). However, is not necessary to performed DNA detection for immunocompetent patients, as serology is sufficient unless the result is indeterminate and an additional test is needed (144), or when the EBV infection is strongly suspected with negative serology results (145). In EBV-associated diseases, the sample of choice differs based on the type of disease. For example, serum can be useful in detecting EBV DNA in Hodgkin’s lymphoma patients as the biology of the disease includes migration of episomal or apoptotic cells derived EBV DNA to the bloodstream. In this specific case, plasma or serum samples are desirable for EBV quantification (124). Similarly, in NPC, cancer cells proliferate in the tissue and uncommonly migrate to the peripheral blood, but cell-free EBV DNA can be detected in the peripheral blood using a serum or plasma sample (124). In contrast, in PTLD, the disease biology involves blast B lymphocytes migration to the bloodstream, accordingly, using a PMNC specimen in preferable (124). Furthermore, the viral load correlates with the severity of the disease in EBV-associated malignancies and lymphoproliferative diseases, and it was found to be a useful prognostic marker (142, 146).

## Conclusion and Future Directions

Epstein–Barr virus is a highly prevalent virus affecting more than 90% of individuals worldwide. Serological diagnosis is widely used in investigating the EBV infection, with VCA-IgG antibodies detection being the best single serological test to indicate previous EBV exposure. Molecular testing is also an important diagnostic tool especially in immunocompromised patients, where serology results may be confusing and unclear due to the incomplete humoral response. However, a combination of both molecular and serological methods would result in early detection of viruses and accurate diagnosis of the infection. Despite the wide number of studies concerning EBV detection, studies investigating the EBV viremia and genetic variability in healthy individuals are still limited, although this virus is transfusion transmissible and linked to PTLDs and a wide range of other malignancies. Estimates of EBV infection are important to give researchers and clinicians accurate data concerning the prevalence of the virus, and consequently, improving the safety of health practices to eliminate the EBV spread, especially in blood banks, and organ transplantation centers where EBV constitutes a life-threatening risk to recipients. In this regard, although recent reports showed that healthy individuals could carry high-risk variants of the LMP-1, which might contribute to cancer development, the majority of the published studies have investigated the genetic variability of the LMP-1 oncogene among cancer patients but not in healthy carriers. For this reason, we believe that understanding EBV molecular epidemiology in different populations and identifying the circulating EBV strains can be an aspect of crucial importance in view of a global preventive approach against all the pathological conditions associated with this virus. Finally, due to the lack of adequate reports from these areas, we believe further studies should be conducted in the Middle East and North Africa regions in order to compare the circulating EBV strains with other regions of the world.

## Author Contributions

GN and MS designed and wrote the first draft of the manuscript with the help of DA-S, GP, and NA. HA-S supervised the whole submission process and addressed the reviewers’ comments.

## Conflict of Interest Statement

The authors declare that the research was conducted in the absence of any commercial or financial relationships that could be construed as a potential conflict of interest.
